# Knowledge Representations: Individual Differences in Novel Problem Solving

**DOI:** 10.3390/jintelligence11040077

**Published:** 2023-04-21

**Authors:** Megan J. Raden, Andrew F. Jarosz

**Affiliations:** Department of Psychology, Mississippi State University, Starkville, MS 39762, USA

**Keywords:** working memory, analogical transfer, reasoning

## Abstract

The present study investigates how the quality of knowledge representations contributes to rule transfer in a problem-solving context and how working memory capacity (WMC) might contribute to the subsequent failure or success in transferring the relevant information. Participants were trained on individual figural analogy rules and then asked to rate the subjective similarity of the rules to determine how abstract their rule representations were. This rule representation score, along with other measures (WMC and fluid intelligence measures), was used to predict accuracy on a set of novel figural analogy test items, of which half included only the trained rules, and half were comprised of entirely new rules. The results indicated that the training improved performance on the test items and that WMC largely explained the ability to transfer rules. Although the rule representation scores did not predict accuracy on the trained items, rule representation scores did uniquely explain performance on the figural analogies task, even after accounting for WMC and fluid intelligence. These results indicate that WMC plays a large role in knowledge transfer, even when transferring to a more complex problem-solving context, and that rule representations may be important for novel problem solving.

## 1. Knowledge Representations: Individual Differences in Novel Problem Solving

Research on novel problem solving (i.e., problems with which the solver is not already familiar) is incredibly diverse, with problem solving being studied in the context of intelligence and reasoning (e.g., Bethell-Fox et al. 1984; Carpenter et al. 1990; Snow 1980), analogical transfer (e.g., Cushen and Wiley 2018; Gick and Holyoak 1980), expertise (e.g., Chi et al. 1981; Novick 1988; Wiley 1998), and even skill acquisition (e.g., Anderson 1987; Patsenko and Altmann 2010). Separately, these domains have addressed different aspects of problem solving (e.g., learning, transfer, knowledge, individual differences, strategy use, etc.) but the lack of communication across those areas has left a large hole in our understanding of how all of these complex processes interact with each other. In particular, studies involving reasoning tasks primarily focus on the role of stable individual differences, such as working memory capacity (WMC; Ackerman et al. 2005; Jarosz and Wiley 2012; Unsworth et al. 2014). In contrast, studies using classic problem-solving tasks or domain-specific tasks (e.g., physics problems) have focused more on strategies and knowledge (Chi et al. 1981; Holyoak and Koh 1987; Novick 1988). Although individual differences in reasoning and problem solving (Kubricht et al. 2017; Ricks et al. 2007; Sohn and Doane 2003, 2004) have been studied from both a working memory and a knowledge perspective, there remain questions about how the two interact.

## 2. Working Memory Capacity and Problem Solving

**Figure 1:**
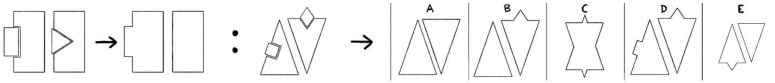
Figural analogy example. *Note.* An example figural analogy item used in the current study. The answer to this item is B.

Much of the early work that investigated individual differences in problem solving used tasks designed to measure fluid intelligence (Gf) because they have a great degree of variability and are intended to be novel, and thus they should not be driven by individual differences in knowledge (Carroll 1993). These tasks were helpful in trying to isolate the non-knowledge-based cognitive processes that contributed to reasoning and problem solving. Early work in this area used tasks such as geometric analogies (Bethell-Fox et al. 1984) or Raven’s Advanced Progressive Matrices (RAPM; Raven et al. 1962) to assess Gf. In many of these tasks, the stimuli are a series of shapes and patterns that change according to rules. The objective of the task is to extract the rules and apply them. An example of a figural analogy problem is shown in Figure 1.

Specific attributes of the stimuli have been shown to increase the difficulty of the problems. These features include how many rules and transformations are included, as well as the number of objects or elements in the problem (Bethell-Fox et al. 1984; Mulholland et al. 1980). The difficulty in the maintenance of these transformations and elements has been ascribed to individual differences in WMC, with storage limits and attentional control being a large barrier to maintaining and manipulating all of the necessary information in memory (Bethell-Fox et al. 1984; Carpenter et al. 1990; Jarosz and Wiley 2012; Mulholland et al. 1980; Primi 2001).

Though there are currently many different theories that postulate different structures for working memory (Baddeley 2000; Barrouillet et al. 2004; Cowan 2005; Oberauer et al. 2003; Unsworth 2016), Unsworth (2016) suggested three components that make up WMC: primary memory, attentional control, and retrieval from secondary memory. Each of these subcomponents may play an important role in the relationship between WMC and Gf (Unsworth et al. 2014), and together explain virtually all of their shared variance. For example, the capacity account (Carpenter et al. 1990) of the WMC and Gf relationship would argue that primary memory allows one to hold the various rules, objects, or goals and subgoals required in a temporary storage space during problem solving. According to the distraction account (Jarosz and Wiley 2012; Wiley et al. 2011), attentional control provides the necessary resources to focus on desired information while ignoring irrelevant or distracting information coming from within or between problems. Retrieval from secondary memory is the process of retrieving previously learned information and can be useful in retrieving rules and transformations that correctly led to previous solutions, a key process in the learning account (Verguts and De Boeck 2002). Each of these components explains unique variance in WMC (Unsworth 2016; Unsworth et al. 2014; Unsworth and Spillers 2010) and appear to uniquely contribute to problem solving and reasoning (Unsworth et al. 2014). However, although these subcomponents can fully explain WMC’s relationship with Gf, WMC does not account for all of the variability found in Gf tasks (Ackerman et al. 2005; Kane et al. 2005; Oberauer et al. 2005).

Although the role of WMC has been heavily studied within reasoning tasks, the mechanistic role it plays during solution and how WMC processes differ from other cognitive processes involved in reasoning tasks remain unclear. Kovacs and Conway (2016) argue that Gf is unlikely to be a unitary construct and, rather, performance on Gf tasks reflects multiple basic processes that are necessary for most Gf tasks. Given that WMC is also comprised of more basic processes and that the shared processes between WMC and Gf do not account for all of the variability in Gf (Ackerman et al. 2005; Kovacs and Conway 2016; Unsworth et al. 2014), other mechanisms must be considered to fully understand individual differences in reasoning and problem solving.

## 3. Individual Differences in Knowledge

The reasoning literature primarily focuses on tasks where the solver has no knowledge, but this is not entirely reflective of problem solving encountered in a real-world setting. In most cases, solvers will have some knowledge pertaining to the problem or will be able to look up information about the problem. Understanding when and how information from memory is used during problem solving is crucial to understanding problem solving as a whole.

### 3.1. Expertise and Representation

Several studies investigating the effects of expertise on problem solving have shown drastic differences in how experts solve problems in their field when compared to novices. The classic study by Chase and Simon (1973) demonstrated an extreme form of chunking, with chess experts recalling four times the information that novices did. Additional research indicated that WMC could help to compensate for a lack of knowledge (Sohn and Doane 2003, 2004), but also that expertise could compensate for a lower WMC, as knowledge becomes more proceduralized and automatic (Patsenko and Altmann 2010).

In addition to having access to more information in memory, experts also show distinct differences from novices in how they represent that information in memory. Experts tend to focus on deeper, more semantically driven representations of problems, whereas novices are more likely to focus on surface features that may not actually be helpful in solving the problem (Chi et al. 1981). Experts’ knowledge representations tend to be highly interconnected, which enables them to move quickly and freely between concepts that are related (Kohl and Finkelstein 2008). Experts are also better at solving problems that are lacking in a clear goal or representation (Simon 1977) by adding in constraints and elaborating on the problem, while novices simply start listing answers or responses to the problem (Voss et al. 1983). Indeed, experts have been shown to create and use goals and subgoals when stuck, whereas novices engage more in exploration (Hershey et al. 1990; Kohl and Finkelstein 2008). Experts’ ability to create goals, Hershey et al. (1990) argue, comes from having plan-like scripts or schemas to help direct them. These highly developed scripts for familiar problems function similarly to productions in the skill acquisition literature, where the scripts and plans increase automaticity. Taken together, this suggests that experts do not simply have more knowledge, but are creating interconnected, abstract representations that benefit them in future problems.

### 3.2. Analogical Transfer

The analogical transfer literature focuses on the ability to generalize previously learned information given in a source problem to a new target problem. Looking at how participants solve a target problem after being given a structurally identical source, multiple studies have found that participants are unlikely to spontaneously transfer solutions (Gick and Holyoak 1980, 1983; Holyoak and Koh 1987; Novick 1988), and even hints do not guarantee transfer (Gick and Holyoak 1983). However, giving participants additional source problems, especially those presented in a spaced-out structure (Gick and Holyoak 1983; Wharton et al. 1994), as well as giving participants a delay between problems (Holyoak and Koh 1987), has been shown to be helpful in building better representations. Findings from the skill acquisition literature also replicate the positive effects of including additional source problems and presenting them in a spaced presentation (Carlson and Yaure 1990). Creating a deeper and richer representation appears to be a key element in transferring knowledge.

Although the representation of the source is crucial for understanding structural similarities between analogs, surface similarities (i.e., how much the problems superficially resemble each other) between the source and the analog also contribute to analogical transfer (Holyoak and Koh 1987) by acting as a cue that there is relevant information in memory. In the absence of surface similarities, solvers must rely on their own internal ability to retrieve a previously learned solution. If solvers represent the source based upon its surface features, then it is less likely that solvers will easily retrieve the source when given a dissimilar target. However, if solvers represent the source in a more general and abstract way (i.e., an emphasis on the deep structure of the source problem, and not surface features), then the target should be a sufficient cue to retrieve the source because the structure, rather than the surface features, is the focus point.

There is some evidence to suggest that the ability to generalize previously learned solutions may actually be driven by WMC or reasoning processes. Kubricht et al. (2017) indicated a positive relationship between analogical transfer and Gf, with additional aids (e.g., providing more source problems) benefitting low-Gf individuals, whereas high-Gf individuals performed well regardless of whether those aids were present. This suggests that high-Gf individuals can more easily form general and abstract representations of the source that can then be transferred more readily. Similar studies have demonstrated the role of diffuse attention in analogical transfer (Cushen and Wiley 2018). Increased attentional resources stemming from WMC may cause fixation on irrelevant details that prevent an individual from noticing that a source and analog are related. In the absence of a more general representation, diffuse attention may help in noticing more remote relationships. Furthermore, the ability to access remote ideas may be helpful in generating more general representations. The results from a study by Cushen and Wiley (2018) demonstrate that performance on the Remote Associates Test does indeed predict analogical transfer, even after accounting for WMC. Furthermore, Remote Associates Test performance also predicted the completeness of someone’s representation (as measured by a summary of the source problem). However, these results are incongruent with Storm and Bui (2016), who found that the propensity to mind wander negatively predicted analogical transfer, even if hints were included. It is possible that performance on the Remote Associates Test taps into an ability to reach remote ideas that is not driven by diffuse attention, or that diffuse attention is only helpful for a portion of the analogical transfer process. Cushen and Wiley (2018) note that once a relationship has been established, the solver must still map the source to the analog. Thus, analogical transfer as a whole may require flexibility in the capacity to use multiple processes.

### 3.3. Learning on Novel Tasks

Despite the fact that many tasks used to study reasoning are intended to provide novel situations, that does not mean that learning cannot occur throughout the task. Several studies involving Gf tasks have demonstrated that participants learn the rules during the task and use them as they approach new problems (Bors and Vigneau 2003; Harrison et al. 2015; Loesche et al. 2015; Verguts and De Boeck 2002). Although participants may be using this information to help them solve problems, the ability to learn these rules well and apply them is more complicated than simply solving a problem correctly. Loesche et al. (2015) provided participants with the rules before completing the RAPM, and although performance did improve, it did not increase to such a point that knowing the rules was sufficient for solving all problems on the RAPM. They noted that this finding was especially sensitive to how well participants initially learned the rules. This is consistent with previously discussed findings in both the skill acquisition literature (Carlson and Yaure 1990; Cooper and Sweller 1987) as well as studies looking at analogical transfer (Gick and Holyoak 1983; Kubricht et al. 2017; Wharton et al. 1994). Interestingly, increases in performance due to rule knowledge do not necessarily change the validity of the tests. Schneider et al. (Schneider et al. 2020; Schneider and Sparfeldt 2021a, 2021b) demonstrated that though learning the rules could increase test performance across several Gf tasks, this increase did not change those tasks’ correlations with other measures of intelligence. This further emphasizes the need to understand how, mechanistically, learning rules influences solution success.

Learning on the RAPM is also sensitive to feedback, as well as exposure to rules throughout the task (Verguts and De Boeck 2002). Additionally, even if participants are given the same problems over multiple sessions, they are inconsistent in solving them. This is true even if they correctly solve the problem the first time (Bors and Vigneau 2003). These results clearly demonstrate that learning and knowledge do play a role in reasoning tasks, but also that previously solving a problem is not a guarantee for solving the same or a similar problem again. An individual’s ability to recognize that previously learned information is relevant, as well as their ability to successfully retrieve the relevant information, are additional processes to consider in conjunction with problem solving or reasoning processes.

## 4. Summary

Problem solving is clearly a complex task that draws upon many different cognitive processes. Individual differences in WMC are a large contributing factor to problem solving success (Ash and Wiley 2006; Carpenter et al. 1990; Sohn and Doane 2003; Unsworth et al. 2014), but there also appears to be room for other processes. Individual differences in knowledge also play a significant role in problem solving (Bors and Vigneau 2003; Chi et al. 1981; Luchins 1942; Wiley 1998). This knowledge can come in the form of expertise or as information that was learned on a previous problem, depending on the task. There is, however, a more complex relationship between knowledge and problem solving. More knowledge does not necessarily mean improved performance (Bors and Vigneau 2003; Gick and Holyoak 1980). Sometimes knowledge can lead to fixation (Luchins 1942; Wiley 1998), sometimes knowledge is not necessary if an individual has high levels of other cognitive abilities, such as WMC (Sohn and Doane 2003, 2004), and sometimes knowledge cannot be used because the individual is unable to transfer it to another problem (Gick and Holyoak 1983; Holyoak and Koh 1987; Kubricht et al. 2017). What seems to be generally beneficial is the production of a general, abstract representation. In addition, the roles of WMC and knowledge are not necessarily completely distinct, and they likely influence each other.

## 5. The Present Study

Although the combination of several literatures has provided information on how knowledge and WMC contribute to and interact during problem solving, there are several research questions that have remained unanswered. Of primary concern are the differential problem-solving processes accounted for by WMC and knowledge. WMC clearly contributes to problem solving, but the mechanisms through which it acts remain under debate (Carpenter et al. 1990; Verguts and De Boeck 2002; Wiley et al. 2011). Because WMC may contribute to knowledge at the encoding stage as well as the retrieval stage, it is important to identify what is breaking down when a solver fails to find or transfer a solution.

To start to isolate knowledge-specific problem-solving processes from general cognitive processes (e.g., attention) in the present study, several changes were made to existing methods in the problem-solving domain. Concerns with analogical transfer stimuli (i.e., using a small sample of fairly difficult problems) were resolved by using a figural analogy task. This ensures that there are multiple target and source problems, as well as making it easier to create problem isomorphs. Additionally, a reasoning task contains more active problem solving than when solely using classical analogical transfer materials. This ensures that there is enough variability in the task that individual differences in problem-solving skill will still be measured.

**Figure 2:**
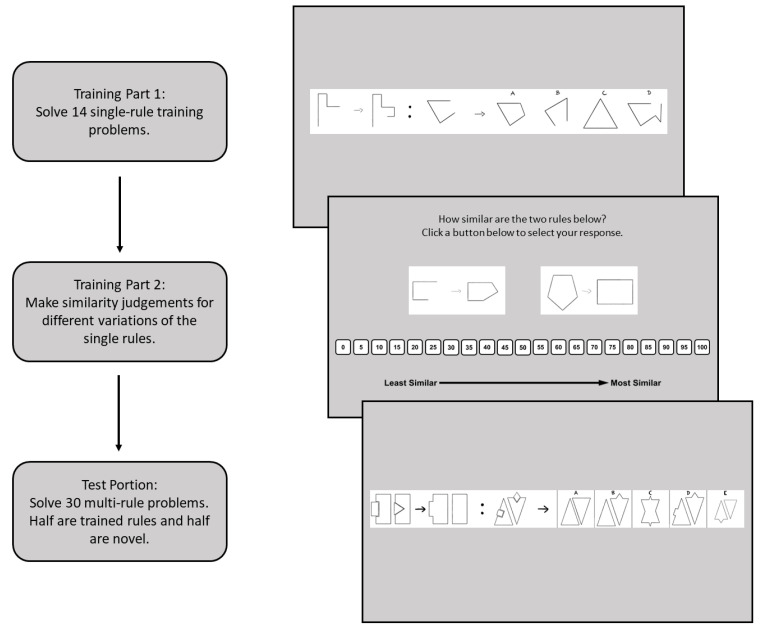
Diagram of figural analogy procedure.

To keep track of knowledge and transfer throughout the task, stimuli were constructed such that all problems could be decomposed into rules. For example, one problem may require two objects to swap locations, a rotation of those objects, as well as a size change. Another problem may only require a rotation and a size change. Problems were considered unique based upon the combination of rules. To test the effects of increased knowledge on problem solving and transfer, participants first learned a select set of individual rules during a training phase, then were tested on more complex problems involving multiple rules. Figure 2 provides an illustration of the general procedure.

To avoid biasing participants towards a particular representation of the rule, participants were not given the rule explicitly. Rather, rules were learned by solving simple problems that contained only one of those rules, (e.g., a problem where the answer is a single size change). The single-rule problems were designed to be solved fairly easily, such that participants could induce the rule on their own. This was accomplished by only using one rule, reducing the visual complexity of items compared to the standard figural analogies task, and reducing the number of response options from five to four. Furthermore, two variations of each rule were shown during the training phase.

During the test portion, participants solved items that included at least two rules. Half of the test items were comprised of only the rules given in the training portion, and the other half were comprised of entirely new rules. If participants were able to successfully transfer the rules learned in the training portion, even in a context where multiple pieces of information must be used, then participants should have higher accuracy on the test items that were comprised of the trained rules when compared to the items that consist of new rules.

Both the expertise literature and the analogical transfer literature have demonstrated that knowledge representations are crucial to knowledge being used, with an emphasis on structure and less emphasis on surface features being particularly beneficial for transferring that knowledge. As such, the representation quality of the rules may be critically important. To measure this, participants were asked to rate numerically how similar they thought two problems were. Participants were shown only the A:B component of a problem rather than the full version with A:B :: C:? so that they could focus on the rule and not on problem solving. The A:B components they saw came from the rules shown in the initial training phase problems. This ensured that participants were making similarity judgements on items for which they had successfully induced the rule previously. Of primary interest was how closely the individual rated slightly different versions of the same rules compared to completely different rules. For example, did the solver rate a 90-degree rotation rule and a 45-degree rotation rule as equally different when compared to a rotation rule and a size-change rule, or did they treat the two versions of the rotation rule as equally similar as two different 90-degree rotation problems? Where participants fell on this spectrum was used to assess their representation, with participants ranging from more general representations (the 90-degree rotation and 45-degree rotation were very similar) to more item-specific, less general representations (the 90-degree rotation and 45-degree rotation were treated as completely separate rules).

If rule representations do indeed help to facilitate retrieval, then more general representations should correlate with higher accuracy on the trained-rule problems. Ratings on the rule representation measure may also correlate with performance on novel-rule problems because the processes that facilitate the generation of general representations may also play a role in other reasoning processes. However, it is expected that there would be a stronger relationship for the trained-rule problems because the novel-rule problems should not be drawing on retrieval processes as much.

The final set of factors to consider is the role of individual differences in WMC and reasoning. It is unclear whether WMC and/or Gf help to develop more general representations, but it is reasonable to expect that WMC and Gf will interact with the training. Because WMC may play a large role in initially learning the rules and then later facilitating the retrieval of those rules during problem solving, WMC should have a stronger relationship with accuracy for the trained items, and Gf should have a stronger relationship with accuracy for the novel items. Furthermore, relationships between the individual differences measures and the rule representation measures can be investigated. Specifically, one question is whether or not the rule representation measure can explain unique variability on the figural analogies task, or if WMC/Gf determine the quality of the representation. If the rule representation measure does uniquely explain performance on the figural analogies task after accounting for WMC and Gf, then this would provide a novel measure that could potentially explain solution transfer. If, however, the knowledge representation measure can be explained by WMC/Gf, then this would provide some further specificity on why WMC and/or Gf explain performance. Whether rule representations correlate or are predicted by WMC or Gf will also be investigated. This will help to further clarify if rule representations are truly unique, share some processes with WMC and/or Gf, or can be entirely explained by WMC and/or Gf.

## 6. Method

### 6.1. Participants

The target sample size for the study, after accounting for participants who fail to complete the tasks correctly or outliers, was set at 200 participants. This was based on simulated analyses demonstrating that 200 participants would be sufficient for detecting moderate effect sizes within a regression model containing a continuous predictor variable, a binary predictor variable, and an interaction term between the two predictor variables. Furthermore, correlations ranging from .3 to .5 begin to stabilize (remain within a window of ±.1 at 80% confidence) at around 212 and 143 participants, respectively, indicating that 200 participants should be sufficient in detecting moderate correlation coefficients as well (Schönbrodt and Perugini 2013). However, because the study was administered entirely online and previously collected online studies at this university have had attrition rates ranging from 40–50%, a new target of 400 participants was set. A total of 418 participants, collected from a pool of Mississippi State University students, completed all tasks in the study. Of those 418 participants, 173 participants failed to meet the inclusion criteria for one or more of the tasks. Twenty-five additional participants were removed for falling outside of 2.5 standard deviations away from the mean on at least one of the tasks or were multivariate outliers, according to Mahalanobis distance (Ghorbani 2019). This resulted in a final sample of 220 participants.

### 6.2. Materials

#### 6.2.1. Modified Figural Analogies Task

The modified figural analogies task is based on the figural analogies test by Lohman and Hagen (2001; Form 6, Level H, Test 8 of the cognitive abilities test). Some of the items from the original task were used in the modified version, but most of the items were created specifically for this experiment. In the standard figural analogies task, participants are shown a set of objects in the form of A:B :: C:? Participants must induce the rule(s) governing the changes from A to B and then apply that rule to the analog, C. Participants select their answer from five response options. An example item is shown in Figure 1.

The modified figural analogies task was split into three parts: a training portion, the Rule-Similarity Judgement Task (RSJT), and a test portion (see Figure 2). For the training portion, participants solved figural analogies items that consisted of only one rule. Thus, participants could learn the rule easily, but it was still through their own induction processes. Then, for the RSJT, participants saw the A:B portion of two figural analogy items and rated how similar they thought the two rules were. The RSJT only used rules that were present in the training portion. Lastly, participants solved a series of figural analogy test items that more closely resembled the items normally shown in the figural analogies task. The test items were comprised of two or three rules, with some items being comprised of rules seen in the training portion and other items being entirely novel. Participants solved both types of problems and the two sets were counterbalanced across participants so that each set of rules was used as the training set and as the novel set at some point.

##### Figural Analogies Training

The participants solved 14 problems in the training portion; 2 problems for each rule. The two problems for each rule were slightly different versions of the rule. For example, for the “size change” rule, one problem was a size change where the object increased in size and the other was a size change where the object decreased in size. This resulted in a total of 14 single-rule items to be used in the training portion for each set, and 28 different versions in total. Participants only solved one set of 14 problems (7 rules) in the training portion because the other set of 7 rules was used to make up the novel rules condition in the test portion.

Prior to data collection, a larger set of rules were piloted. Some of the rules originated from the original figural analogies task, whereas others were generated specifically for this study. Pilot data indicated that the final set of 14 rules were all treated as distinct by participants and that the expected interpretation of the rule matched with participants’ verbal descriptions of the rules. For example, many participants described a rule wherein an object duplicates as “double”, “duplicating”, “multiply by 2”, “copy shape” or some other variation. These types of responses were consistent for the other rules, wherein there was some variation in the words participants chose, but the overall explanation aligned with the expected interpretation. The full list of rules, and which set they belonged to, can be found in Appendix A. Participants were given an unlimited amount of time to solve the training items. Because the validity of the RSJT and the effect of training both depended on participants successfully inducing the rules, participants were removed from analyses if they missed more than 4 problems in the training portion. Of the 418 participants that completed all tasks, 89 failed to meet the inclusion criteria for the training portion.

##### Rule-Similarity Judgement Task (RSJT)

After solving the 14 training items, participants made similarity judgements on the rules using a scale that ranged from 0 to 100, in intervals of 5. They were shown all of the items in a random order and had unlimited time to make the judgement.

**Figure 3:**
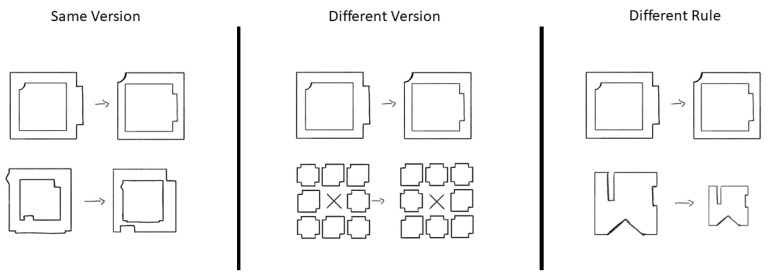
RSJT comparison stimuli example. *Note.* The same version comparison example includes two swap rules, the different version comparison includes two slightly different swap rules, and the different rule comparison includes a swap rule and a size change rule.

Participants were shown only the A:B portion of the problem so that they could focus on only the rule. The rules used in the similarity judgement portion were the same rules used in the training portion, so participants had already demonstrated their ability to induce the single rule, which is necessary for making a comparison. Each comparison could be categorized into three conditions: the exact same version of the same rule (“same-version comparison”), two different versions of the same rule (“different-version comparison”), and different versions of different rules (“different-rule comparison”). Examples of the stimuli are provided in Figure 3. An example of the same-version comparison condition would be a comparison between two different 90-degree rotation items. An example of the different-version condition would be a 90-degree rotation item and a 45-degree rotation item comparison. An example of the different-rule condition would be a 90-degree rotation item being compared to an increase in size; the size-change rule.

Pilot analyses indicated that participants were responsive to the different types of comparisons, with the average rating for same-version comparison being the highest (*M* = 82.33, *SD* = 28.55), and the average rating for different-version comparison (*M* = 56.56, *SD* = 35.95) being between same-version and different-rule comparisons (*M* = 18.32, *SD* = 25.06). Furthermore, there were almost no two rules that were treated as being too similar, except for three different-rule comparisons that had an average rating over 30. These rules, however, were separated into different groups to prevent any issues that might have been caused by their similarities. Finally, subtle differences in the types of rules (quantifiably different rules, such as rotation, vs. qualitatively different rules, such as color change) did not systematically contribute to participants’ similarity judgements.

**Figure 4:**
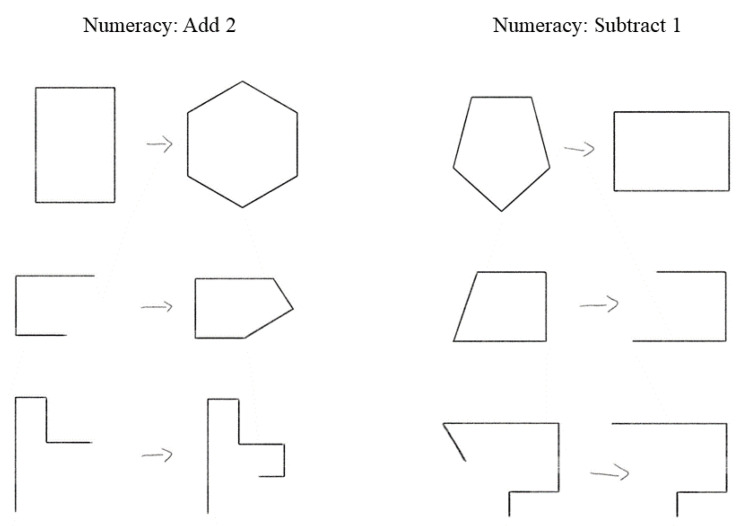
Numeracy A:B items. *Note.* Examples of the RSJT items used for the numeracy rule.

In order to provide a sufficient number of comparisons, six A:B items were created for each rule, including three for each rule version (i.e., three 90-degree rotation items and three 45-degree rotation items). The six A:B items were drawn with enough variation that there were no isomorphs during the same-version comparisons. However, all items were drawn with most shapes being square-like to encourage participants to look at the rule and not simply make a similarity judgement based upon surface features. An example of the six items for the numeracy rule is shown in Figure 4. With six items, six same-version comparisons were shown (three per rule), six different-version comparisons were shown (more combinations were technically possible, but six comparisons were randomly selected), and twelve different-rule comparisons per rule were shown. Although more combinations are possible, only two comparisons per rule (one per version) were selected to be matched with other rules. This resulted in a total of 126 comparisons: 42 same-version comparisons, 42 different-version comparisons, and 42 different-rule comparisons. All comparisons were shown in a random order across all participants. Prior to starting the similarity judgement portion, participants were given three practice trials, one per comparison type, so that they could adjust to the procedure and the types of comparisons they would be making.

To ensure that participants were completing the similarity judgement task appropriately and not simply selecting random responses, a *t*-test was calculated for each participant to determine if they were treating the same-version rule condition differently than the different-rule condition. Given that these fall on two extremes, participants should rate these differently, and thus this served as a manipulation check. Of the 418 participants that completed all tasks, 57 failed to meet the inclusion criteria for the RSJT.

##### Figural Analogies Test

For the test portion, participants completed a total of 30 figural analogy problems. Half of the problems were comprised entirely of rules used in the training portion and the other half were comprised of novel rules. However, what constituted trained rules or novel rules depended on which set of rules the participant received in the training portion, with the two set of rules counterbalanced across participants. Pilot analyses from a larger sample of figural analogy problems were used to create the sample of thirty problems to ensure that the two sets of problems were equal in difficulty. The figural analogies test problems consisted of two or three rules (seven two-rule problems for Set A and nine for Set B, with the remainder of the set being three-rule problems), with some problems consisting of entirely distinct rules, whereas other problems used two versions of the same rule in a problem (paired-rule items). The paired-rule items were introduced to increase the number of problems used in the figural analogies test portion while still ensuring that no two problems used the same combination of rules. Furthermore, the practice of using two rules conditional on some other factor (such as the size of the object) is a technique used in the RAPM (Raven et al. 1962) and therefore has precedent for being used in a Gf task. The distinct-rule items could be comprised of two or three rules, but the rules were always unique (e.g., color change, size change, and rotation). The paired-rule items could also consist of two or three rules, but always included two rules that were actually two versions of the same rule. For example, one problem may have a 90-degree rotation for the larger object and a 45-degree rotation for the smaller object. If the paired-rule item consisted of three rules, then the third rule was an additional distinct rule. Pilot analyses indicated that the paired-rule items did not differ in difficulty when compared to the distinct-rule items. Set A did include more paired-rule items than Set B (nine and seven, respectively), but this was a result of prioritizing balanced difficulty across the two sets.

Participants were shown the 30 problems in a random order and were given a maximum of 60 s to solve the problem. If they had not selected a response after 60 s, they were moved to the next problem and that item was marked as incorrect. To encourage accurate performance on the task, a 20 s penalty screen appeared if participants selected a response in less than 5 s and it was incorrect. The screen notified the participant that they selected their response quickly and encouraged them to make sure that they were performing the task correctly. To serve as a manipulation check, the example figural analogies item shown in the instructions (A:a :: R : ?) was drawn to resemble the figural problems more closely (adding triangles and rectangles around the letters) and placed randomly in the figural analogies test portion. Participants that failed to solve the manipulation check item (52/418) were removed from analyses.

#### 6.2.2. Working Memory Capacity

##### Automated Operation Span 

The automated operation span (Unsworth et al. 2005) is a complex span working memory task that tests an individual’s ability to remember letters while solving simple math problems in between the presentation of the letters. For the math portion, participants were shown a simple math problem followed by a number. They had to determine if the number was the answer to the previous problem or not. They were then shown a letter that was to be recalled at a later time. After several iterations, participants were asked to recall the letters in the sequence that they saw them in. Participants completed 2 blocks, with each sequence length, ranging from 3 to 7 letters, being presented once per block. The task was scored using the partial scoring method, where a participant’s total is comprised of the total correct letters recalled in the correct position of the sequence. Participants completed a shortened version (Foster et al. 2015) of the task (two blocks instead of three) to try and reduce fatigue because participants completed all tasks online. Participants were removed if they failed to score at least 80% accuracy on the math portion (40/418).

##### Automated Symmetry Span 

The automated symmetry span is very similar to the automated operation span, but it uses visual stimuli rather than verbal stimuli (Unsworth et al. 2009). Instead of math problems, participants determine if an image is symmetrical or not. Then, they are shown a 4 × 4 grid with a single red square filled in. After several iterations, of which range from 2 to 5 to be remembered red squares, participants recall the location of the red squares in the sequence they were presented. Participants completed two blocks (Foster et al. 2015) and the task was scored using the partial scoring method. Participants were removed if they failed to score at least 80% accuracy on the symmetry portion (59/418).

#### 6.2.3. Gf Measures

##### Paper Folding Task 

**Figure 5:**
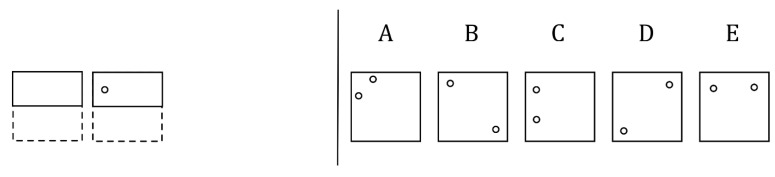
Example of paper folding item. *Note.* A paperfolding example from Ekstrom et al. (1976).

The paper folding task (Ekstrom et al. 1976) shows participants a piece of paper that is then folded several times. A hole is then punched through the folder paper and participants must determine what the unfolded paper would look like. Participants were shown a total of 20 items, with 3 min to complete the first 10 and another 3 min to complete the second set of 10. An example item is shown in Figure 5. Because it was easy to click through this task and the study was administered online, participants were excluded from all analyses if their average response time across all items was less than two seconds (25/418).

##### Letter Series 

The letter series task (Kotovsky and Simon 1973) requires participants to identify a pattern in a sequence of letters and provide what the next letter in the sequence would be according to that pattern. For example, participants might be given ‘ababababa’, with the correct answer being ‘b’. Participants solved 15 items with no time limit for the task. Similar to the figural analogies test portion, however, participants were shown a 20 s time penalty screen if they selected their response in less than 5 s and it was incorrect. To find participants that were clicking through the task, the problem “abcde” was added as the tenth item (changing the total to 16 items). However, after data collection was completed, it was realized that “abcde” could be solved with multiple answers, so instead participants were excluded from analyses if their accuracy was less than three. Three was chosen because the first two items of the letter series task are extremely easy and most participants did solve the “abcde” item correctly, suggesting that most participants genuinely doing the task should be able to solve at least three problems correctly. Of the 418 participants that completed all data, 66 failed to meet the inclusion criteria for the letter series task.

### 6.3. Procedure

Participants completed all tasks in a single session, entirely online. Participants were told prior to starting the study that they must complete the study in one sitting so they should only begin when they are ready. Participants were given a final warning screen before starting the study to not begin unless they were ready. Participants completed the modified figural analogies task first, followed by the automated operation span task, the symmetry span task, the paper folding task, and the letter series task. All tasks were completed in this order, with a short one-to-two-minute break provided between each task.

## 7. Results

Composite scores were generated for the WMC and Gf measures by taking performance on the two tasks (operation span and symmetry span for WMC and paper folding and letter series for gf), *z*-transforming the scores, and then taking the average of the two tasks^1^. For the modified figural analogies task, three rule-representations scores were generated from the RSJT, one score for each type of comparison (i.e., the same rule and the same version of the rule, or “same version”; the same rule but a different version of the rule, or “different version”; or a different rule and different version, or “different rule”). These were calculated by first recentering all the scores for each participant such that their minimum score became −1 and their highest score became 1. The recentering was performed across all of their judgement scores and was not split by type of comparison. This was carried out to account for participants using the scale differently^2^. Next, the average score was taken for each type of comparison for each participant, producing three scores: their average same-version comparison score, different-version comparison score, and different-rule comparison score. The primary measure of focus was their average score for the different-version comparisons. If participants have a positive value for their different-version score from the RSJT, then this would indicate that they are treating the different-version items similar to the same-version items, suggesting their representation of that rule is more general and that they are not treating different versions as entirely different rules. Conversely, if their different-version score is negative, then this would indicate that they are treating the different-version rules as separate rules.

### 7.1. Summary Statistics and Correlations

All summary statistics are included in Table 1 and the correlations between all tasks are shown in Table 2. The RSJT scores were split by the two stimulus sets in Table 1 so that reliability could be calculated for each set. Reliability was calculated using Cronbach’s alpha at the item level for the figural analogies test items, letter series, and the RSJT same-version and different-version scores (averaged across each rule, treating it as an item). Split-half reliability was used for paper folding and the RSJT different-rule scores. Split-half reliability was used for the RSJT different-rule scores because they could not be averaged by rule, as two rules were shown in the different-rule comparisons. Parallel-forms reliability was used for operation span and symmetry span.

### 7.2. Predicting Figural Analogies Accuracy with Training, RSJT Scores, WMC, and Gf

The first set of analyses investigated whether accuracy on the figural analogies test items changed as a function of training, the participant’s rule representation as measured by the RSJT different-version scores, and the WMC and Gf measures. To determine this, generalized linear mixed-effects models (GLMM) were used to predict item accuracy on the test items with the fixed effects hierarchically introduced. A logit link function was used to predict the binary accuracy data. The maximum random effects structure included random intercepts for subject and item, with random slopes added for training for both subject and item. The general procedure for the models was to include all fixed effects and the maximum random effects structure initially and to then gradually reduce the complexity of the random effects structure if the model failed to converge or was singular (Bates et al. 2015; Matuschek et al. 2017). The structure could be reduced by removing correlation parameters or removing random effects that failed to explain a significant portion of variance (i.e., if variance was not greater than zero at α = 0.20). All binary predictors were coded using sum contrast coding (−1, 1) and all continuous predictors were *z*-transformed.

**Figure 6:**
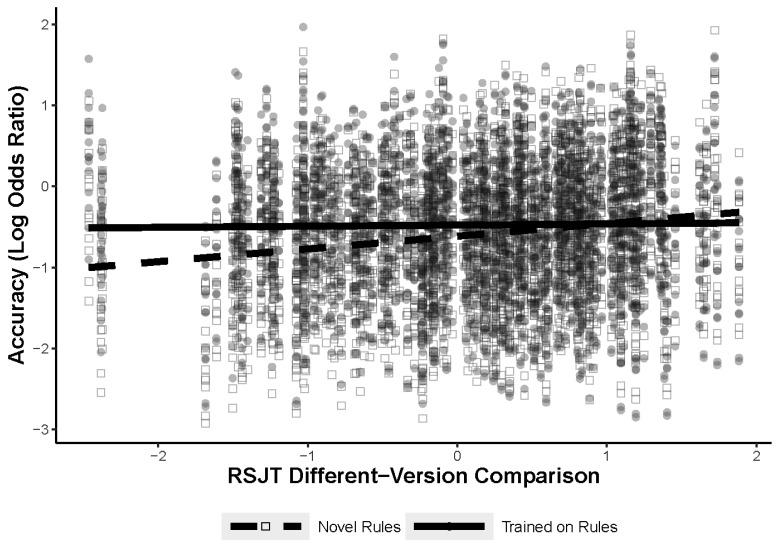
Interaction between RSJT different-version scores and training. *Note.* The plot was generated using the predict function in R to generate log odds ratio accuracy data based upon Model 1 (Table 3). The data points represent item level data for all participants and the linear slopes were generated with the geom_smooth function in ggplot. The circles represent trained-rule items and the squares represent novel-rule items.

The first model predicted figural analogy accuracy with fixed effects for the training (items comprised of previously seen or novel rules) and the participants’ RSJT different-version score with a test for an interaction between RSJT different-version scores and training. The results of the model, Model 1, are shown in Table 3. There was a significant interaction between the training manipulation and RSJT different-version scores, shown in Figure 6; however, the relationship was in the opposite direction of what was predicted. The interaction illustrates a small relationship between RSJT different-version scores and accuracy, but only when participants are solving problems comprised of novel rules. The RSJT scores did not predict accuracy at all for the trained items. It is worth noting that only three participants fell outside of −1.7 standard deviations away from the mean for the RSJT different-version scores but were within −2.5 standard deviations. The removal of these participants did not change the results of the model.

The next analysis introduced the WMC and Gf composite measures into the GLMM. WMC and Gf were added into the models to determine if rule representations accounted for unique variance or if they could be explained by other processes. Both WMC and Gf measures were included because prior work investigating analogical transfer has shown that Gf may contribute to building representations (Kubricht et al. 2017), but WMC was not accounted for in that study.

WMC and Gf were added on top of the RSJT different-version scores, with WMC and Gf also tested for interactions with training. Because the inclusion of interaction terms can make it difficult to interpret main effects, nonsignificant interactions were removed for the final model, Model 4. However, the WMC and Gf models with all interaction terms are also included. All models’ results are shown in Table 3. For WMC, shown in Model 2, there was no significant interaction with training, though WMC did predict accuracy on the figural analogies task. For Gf, in Model 3, there was also no significant interaction, but Gf did predict accuracy on the figural analogies task. The interaction between RSJT different-version scores and training did remain significant after including both Gf and WMC, indicating that the RSJT scores were measuring something unique from Gf and WMC.

#### 7.2.1. Interim Discussion: Task Learning

The interaction between RSJT different-version scores and training was in an unexpected direction. These results may indicate that processes that would normally be beneficial in solving figural analogies problems become less important or less likely to be used when participants have been trained on the rules. This is similar to findings in the expertise literature, where dependence on other cognitive processes, such as working memory, decreases as expertise increases. The lack of an interaction between WMC and training was also unexpected, given prior work showing that the relationship between performance on Gf tasks and WMC changes depending on whether items are comprised of novel or previously seen rules (Harrison et al. 2015; Loesche et al. 2015; Wiley et al. 2011). However, it has also been noted that participants learn throughout a task, even if all of the rules are novel, and that the frequency of rules used can bias participants’ expectations and use of rules (Verguts and De Boeck 2002). Therefore, a post hoc analysis was conducted to investigate the relationship of WMC and training on accuracy while also accounting for learning throughout the task.

#### 7.2.2. Task Learning Post Hoc Analysis

**Figure 7:**
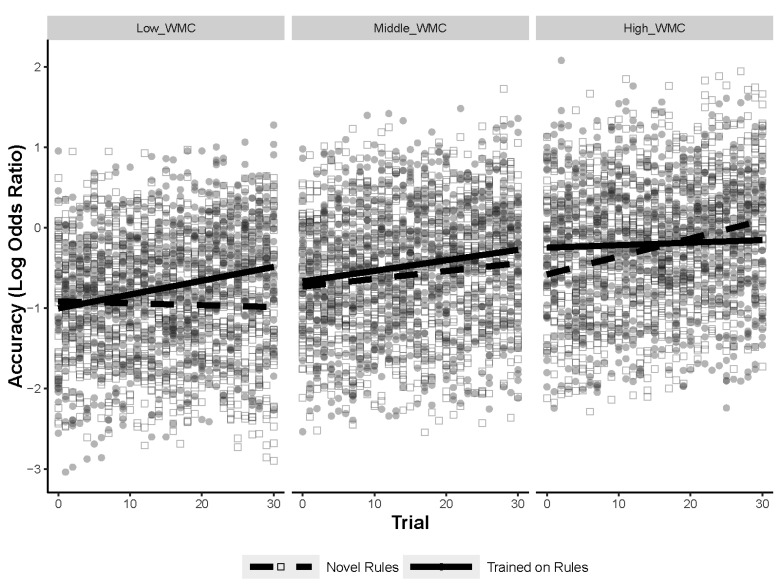
Interaction between WMC, training, and trial. *Note.* The plot was generated using the predict function in R to generate log odds ratio accuracy data based upon the model in Table 4. The data points represent item-level data for all participants and the linear slopes were generated with the geom_smooth function in ggplot. The model was analyzed with WMC as a continuous predictor but a tertiary split was used to plot the data. The circles represent trained-rule items and the squares represent novel-rule items.

Learning throughout the task was assessed using trial number, so a separate analysis tested for a three-way interaction between WMC, training, and trial number, while predicting accuracy on the figural analogies task. The results of the GLMM are shown in Table 4. The three-way interaction was significant (*p* = .003), as shown in Figure 7. Post hoc comparisons demonstrated that WMC and trial continued to interact for the novel-rule items (*p* = .021) but not for the trained-rule items (*p* = .07). However, there was still a main effect of WMC (*p* < .001) and trial (*p* = .02) for the trained-rule items (even with the interaction term removed). The models for the post hoc comparisons can be found in Appendix A. The results of the three-way interaction and the post hoc comparisons suggest that high-WMC individuals improve on the novel-rule items over time whereas the low-WMC individuals do not. Although the interaction term between WMC and trial was not significant for the trained-rule items, the main effects of WMC and trial indicate that high-WMC individuals were more likely to solve trained-rule items, but both high and low-WMC individuals improved on the trained-rule items over time. Notably, there were no significant interactions with Gf for training and trial number, so these results appear to be unique to WMC.

### 7.3. Predicting RSJT Different-Version Scores

After investigating the relationship between memory retrieval and accuracy, a closer look at the rule representation measure was conducted to determine if WMC and/or Gf could explain the ability to generate more general rule representations. The second analysis used a linear mixed-effects model (LMM) to predict the RSJT different-version scores for each rule, with the WMC and Gf measures used as predictors. The random effects structure included random intercepts for subject and rule. The *p*-values were generated using the lmertest package in R. The results of the model can be found in Table 5. Neither Gf nor WMC predicted the RSJT different-version scores.

#### 7.3.1. Interim Discussion: Further Investigating the RSJT Scores

The lack of a relationship between RSJT different-version scores and figural analogies accuracy on trained items, as well as the fact that RSJT same-version scores appeared to better correlate with figural analogy accuracy, prompted a deeper inspection of the RSJT scores and what they may actually measure. The original intention with the RSJT was to use the different version comparison scores only, because it was assumed that most participants would rate the same-version comparisons as very close to 1 and the different-rule comparisons as very close to −1, reducing those measures to be at the ceiling or at the floor. Although same-version comparisons were rated the highest on average and the different-rule comparisons were rated the lowest on average, there were no ceiling or floor effects, thus making these other RSJT scores potentially meaningful.

It is also possible that different processes are beneficial when solving an item that has two very similar rules included in a single item. The two types of figural analogy items, distinct-rule items (items comprised of all unique rules) and paired-rule items (items where two versions of the same rule appear in the same item), were originally developed to help control for difficulty across the two sets and to allow for a larger set of items by repeating rules. This requires the solver to be open to different variations of a rule and to not be overly fixated on a single element of the rule. Yet, it also requires that the solver does not over-generalize such that they cannot distinguish between the two highly similar rules. As such, the following analyses explore both the other two RSJT measures and their impact on different types of items.

#### 7.3.2. Further Investigating the RSJT Scores Post Hoc Analyses

The first analysis sought to determine if each of the three RSJT scores measure different constructs. The three measures were used as simultaneous predictors in a GLMM predicting figural analogies accuracy. The results are shown in Table 6. The RSJT same-version and RSJT different-rule measures did uniquely predict accuracy, but the RSJT different-version did not predict accuracy, congruent with prior analyses.

**Figure 8:**
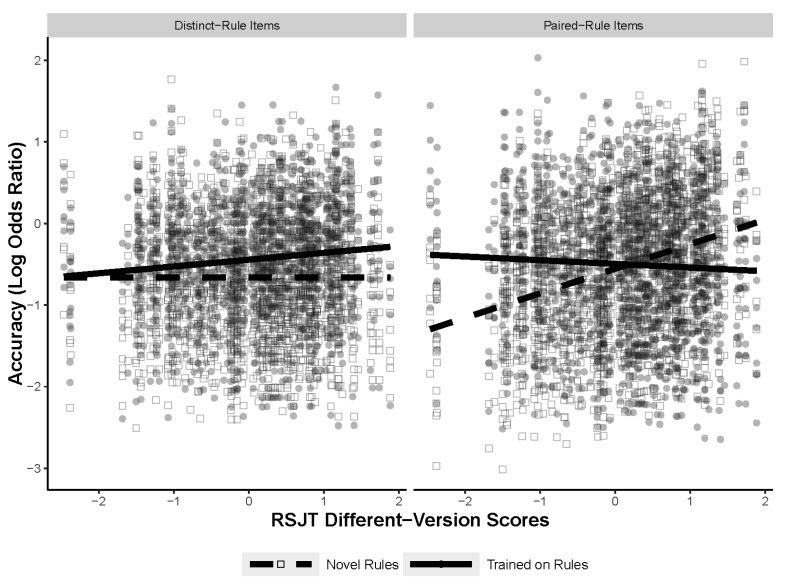
Interaction between RSJT different version scores, training, and item type. *Note.* The plot was generated using the predict function in R to generate log odds ratio accuracy data based upon the different-version model in Table 7. The data points represent item-level data for all participants and the linear slopes were generated with the geom_smooth function in ggplot. The circles represent trained-rule items and the squares represent novel-rule items.

The next analysis explored whether the type of figural analogy item being solved contributed to the predictiveness of the RSJT measures. To test if the type of problem mattered, three mixed-effects models were run, each with a different RSJT measure. The models tested for a three-way interaction between the RSJT measure, training, and the type of figural analogy item (distinct or paired) with random intercepts for subject and item, and random slopes for training for both subject and item. The final models are shown in Table 7. The type of figural analogies item only interacted with the RSJT different-version measurements. Figure 8 shows the three-way interaction between RSJT different version, training, and type of figural analogy item. Post hoc comparisons specified that the significant differences in slopes were between the novel paired-rule items and the trained paired-rule items, as well as the novel paired-rule items and the novel distinct items. All the models for the post hoc comparisons can be found in Appendix A. The results indicate that how an individual rates different versions of the same rule does predict accuracy on the figural analogies task, but only for novel paired-rule items. Furthermore, this interaction was not significant with the RSJT same-version or different-rule measures, suggesting that this is particular to the different version measure. Notably, the results found with the three-way interaction likely explain the effect found in the first analysis, with RSJT different version interacting with training. Finally, the lack of a main effect of item type, as well as a two-way interaction between training (namely in the same version and different rule models that do not include the three-way interaction term), indicate that the type of item did not contribute to overall accuracy and is also not responsible for any of the training effects.

#### 7.3.3. Interim Discussion: Measuring Different Version Relative to Same Version and Different Rule

The original different-version measure represented an individual’s average score for the different-version comparisons after rescaling all of their scores from −1 to 1. Although this method also provides measures for same-version and different rule, it fails to account for participant and item effects, it does not take into account the spread of an individual’s ratings (only the average), and it does not provide any information on how an individual rates different-version comparisons relative to the same version or different rule. For example, some participants may rate different-version rule comparisons as similar, but they may also rate different-rule comparisons as similar, suggesting that they have a general propensity toward calling all rules similar. Alternatively, participants may view same-version rules as being equally as distinct as different-version rules. Although all participants included in the sample needed to have a significant difference between the same-version and different-rule conditions to be included, the current measures do not provide information on how large of a difference there exists between the same-version and different-version comparisons, as well as the different-version and different-rule comparisons.

#### 7.3.4. Relative Rule Post Hoc Analysis

To better address ratings of different-version rules relative to the other two rule comparison types, new measures were generated using a mixed-effects model. The linear mixed- effect model predicted raw similarity judgement ratings with type of comparison (same version, different version, different rule) as a fixed effect. The random effects included random intercepts for item and participant, with random slopes for type of comparison for each participant. Type of comparison was dummy-coded as two variables, same version–different version and different rule–different version, with same version coded as 1 for the same version–different version measure and different rule coded as 1 for the different rule–different version measure, and everything else coded as 0. The random slopes for the same version–different version and different rule–different version measures for each participant were extracted and used as predictors in a series of analyses. The same version–different version and different rule–different version measures represented the difference between the two types of comparisons. Individuals with higher (positive) values for the same version–different version measure on average rated same-version comparisons as higher than different-version comparisons, whereas individuals with lower (negative) values rated different-version comparisons as being closer to same-version comparisons. For the different rule–different version measure, individuals with larger (positive) values rated different-version comparisons as closer to different-rule comparisons, whereas individuals with lower (negative) values rated different-version comparisons as different from different-rule comparisons. Because the new measures produced some outliers for those measures (beyond 2.5 standard deviations away from the mean), some participants were removed for subsequent analyses. This resulted in a final sample of 215 participants. The correlations between the new RSJT measures, the old RJST measures, figural analogies accuracy, WMC, and Gf are in included in Table 8.

**Figure 9:**
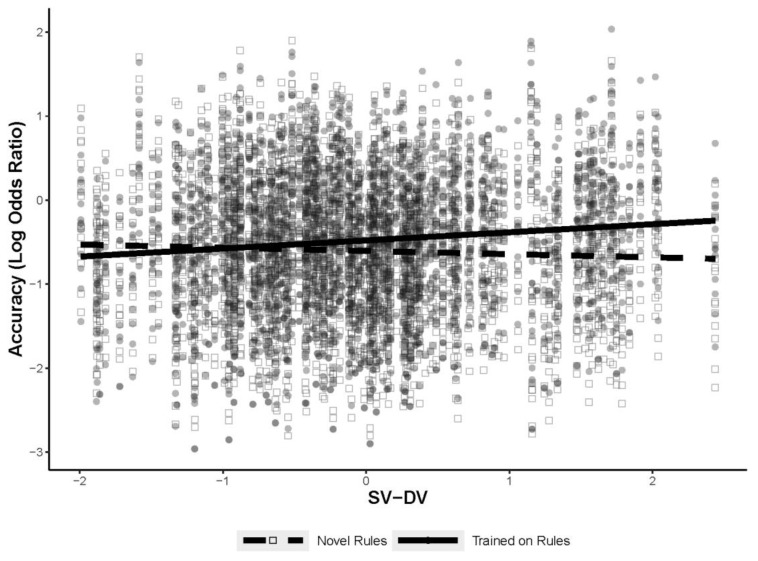
Interaction between different rule–different version and the training manipulation. *Note.* The plot was generated using the predict function in R to generate log odds ratio accuracy data based upon the model in Table 9. The data points represent item-level data for all participants and the linear slopes were generated with the geom_smooth function in ggplot. The circles represent trained-rule items and the squares represent novel-rule items.

The first analysis used a GLMM to predict figural analogy accuracy as a function of the two new measures, the training manipulation, WMC, and Gf, with a test for an interaction between the two new measures and the training manipulation. The random effects structure was identical to previous analyses, with random intercepts for subject and item, with random slopes added for training for both subject and item. The same version–different version and different rule–different version measures were *z*-transformed prior to including them in the model. The final model is shown in Table 9. There was a significant interaction between the same version–different version measure and training, but not for the different rule–different version measure. The interaction is shown in Figure 9. The interaction indicated a positive relationship between the same version–different version measure and figural analogies accuracy for the trained items only. Thus, individuals that tend to rate different-version comparisons as different from same-version comparisons show improved accuracy for the trained-rule items. These results are the opposite of what was found previously when just using the RSJT different version measure. Although there was no significant interaction with the different rule–different version measure, it did predict figural analogy accuracy as a main effect, with a larger difference between different version and different rule scores corresponding with higher accuracy on the figural analogies test items.

**Figure 10:**
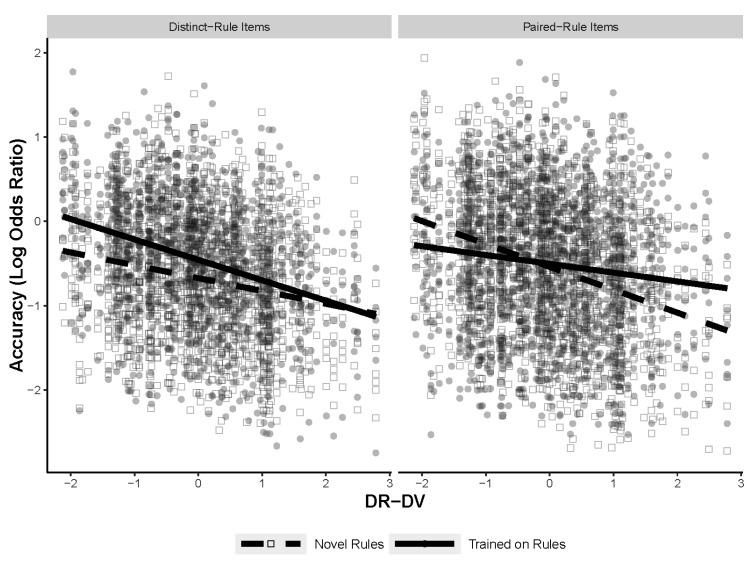
Interaction between different rule–different version, training, and item type. *Note.* The plot was generated using the predict function in R to generate log odds ratio accuracy data based upon the different rule–different version model in Table 10. The data points represent item-level data for all participants and the linear slopes were generated with the geom_smooth function in ggplot. The circles represent trained-rule items and the squares represent novel-rule items.

The next analysis tested for interactions between the two new RSJT measures, the training manipulation, and item type (distinct vs. paired rules). The final models are shown in Table 10. There was a significant interaction between all three measures when different rule–different version was included, but not when same version–different version was included. The results of the interaction are shown in Figure 10. Post hoc comparisons indicated that the only significant difference in slopes was between the novel paired-rule items and the trained paired-rule items. The full models for the post hoc comparisons are located in Appendix A. These results are largely congruent with previous results showing a significant three-way interaction between different-version scores, training, and item type. Compared to the previous analyses, the current model specifies that not only is it higher different-version scores that predict accuracy on novel paired-rule items, but that higher different-version scores relative to different-rule scores predict accuracy on novel paired-rule items.

## 8. Discussion

### 8.1. Knowledge Representations and Transfer

The present work examined the unique and interacting impact of processes driven by WMC and knowledge representations on success during reasoning. The potential for more thorough knowledge representations to better facilitate transfer is not a novel concept (Anderson 1987; Gick and Holyoak 1983; Holyoak and Koh 1987; Wharton et al. 1994) and neither is the contribution of knowledge to performance on Gf tasks (Bors and Vigneau 2003; Harrison et al. 2015; Loesche et al. 2015; Verguts and De Boeck 2002). Similarly, the role of WMC during reasoning has been explored previously (Carpenter et al. 1990; Verguts and De Boeck 2002; Wiley et al. 2011). Where the present study breaks new ground is in looking at these mechanistically, during a Gf task.

Although both knowledge representations and WMC did predict success during the figural analogies task, the results did not align with a priori predictions. It was assumed that the presence of general rule representations would better facilitate transfer, as measured by the RSJT different-version scores. However, although RSJT different-version scores did interact with the training manipulation, they only predicted accuracy on novel items, not the trained items. This was opposite of what was expected. The original reasoning was that generalized rule representations would facilitate retrieval, but it is possible that the training or even the RSJT itself helped most participants conceptualize the rules in a way that was useful, thus leaving retrieval of the trained rules to depend on other processes, such as WMC. That being said, the analyses that included same version–different version measure did provide some support for rule representations facilitating retrieval. The same version–different version measure only predicted accuracy for the trained-rule items, not the novel-rule items. However, it also showed a positive relationship, suggesting that individuals who treat different-version comparisons as being less similar than the same-version comparisons had improved accuracy on the figural-analogy-trained items. This is also contrary to what was expected. Although these interactions differed from expectations, they did remain significant once WMC and Gf were introduced into the models. This indicates that the RSJT can uniquely explain variance on novel and trained figural analogies items beyond what Gf and WMC can explain.

The interaction between training and RSJT different-version scores also increased when accounting for the type of figural analogy item (distinct vs. paired rules). It is likely that the initial two-way interaction between training and RSJT different-version scores is driven by the three-way interaction with figural analogy items, and that in truth RSJT different-version scores really only predict novel paired-rule items. However, the relationship was positive, congruent with the initial hypotheses of the study. Mentally representing two versions of the same rule as closely similar, as opposed to treating them as distinct rules, corresponded with higher accuracy on novel problems where two versions of the same rule were included. Furthermore, the different rule–different version analyses indicated that it may be more important that individuals treat different-version comparisons as different from different rules than it is for different-version comparisons to be treated as similar to same-version comparisons. Notably, the fact that the RSJT different-version scores, as well the different rule–different version scores, only predicted novel items suggests that individuals with a propensity to treat different versions of the same rule as more similar, but still as separate rules, may have an easier time inducing related, but slightly different rules when they are exposed to them for the first time. It is likely that the training or the RSJT helped participants induce and develop representations to a point where individual differences in RSJT different-version scores no longer mattered for solving the trained-rule problems.

Unlike the RSJT different-version scores, WMC did appear to explain transfer, showing interactions with the training manipulation when accounting for learning across the task. Both high- and low-WMC individuals improved on trained rules over time, but high-WMC individuals were more likely to solve trained items correctly than low-WMC individuals. Furthermore, high-WMC individuals improved on the novel-rules items as they progressed, with performance increasing as they solved more items. Low-WMC individuals did not show this benefit, appearing to struggle on novel items throughout the task. It is possible that high-WMC individuals improved on the novel items by learning the rules and using them later in the task (Harrison et al. 2015; Loesche et al. 2015; Verguts and De Boeck 2002), or they may have simply improved in solving novel problems, learning new strategies or becoming more comfortable with the task (Klauer et al. 2002; Tomic 1995). The low-WMC individuals did not improve on the novel problems over time, suggesting that they struggled to induce the rules at all, or that they may need substantially more practice before they begin to benefit from learned rules.

These results are congruent with prior work in multiple ways. First, the fact that high-WMC individuals were able to benefit from trained rules more than low-WMC individuals indicates that WMC is important for retrieving previously learned information (Loesche et al. 2015; Unsworth 2016). Second, only the high-WMC individuals improved on novel items over time, whereas both low- and high-WMC individuals improved on the trained items over time, indicating that WMC also contributes to learning throughout the task (Harrison et al. 2015; Unsworth 2016). Finally, because the high-WMC individuals were generally better at solving novel items and low-Gf individuals did not improve at the novel items over time, WMC seems important for initially inducing the rules (Carpenter et al. 1990). Although these results support several explanations for the role of WMC in Gf tasks, it is worth noting that the findings only became apparent after testing for the three-way interaction with trial number. Furthermore, these results fail to replicate studies showing a stronger relationship between WMC and repeated rules (items wherein the combination of rules has been seen previously) on the RAPM (Loesche et al. 2015; Harrison et al. 2015) or studies showing the opposite, where WMC better predicts novel (unique combinations) RAPM items (Wiley et al. 2011). Taking into consideration learning across the task, as well as accounting for individual rules rather than just unique combinations of rules, may explain the mixed findings on the role of WMC in Gf tasks.

Finally, it is worth noting that WMC may play a large role in how well the rules are initially learned. Previous work with analogical transfer has found that Gf may explain the ability to develop more general representations that are more easily transferred (Kubricht et al. 2017). However, WMC and Gf are highly correlated and so it is possible that the high-Gf individuals in Kubricht et al.’s study did not benefit from the training manipulation because they were also high in WMC, thus learning the source problem better. This would explain why WMC is shown to explain transfer in the current study and not Gf. Yet, it is worth noting that Cushen and Wiley (2018) found that WMC only explained the completeness of an individual’s summary of the source problem when performance on the Remote Associates Test was not accounted for. Thus, although WMC may be important for retrieving learned rules, other processes may still be important in helping to generate more general representations, especially in cases of more complex learned information.

### 8.2. The Rule-Similarity Judgement Task

The original intention with the RSJT was to only use the different-version scores, but the same-version and different-rule scores ultimately did provide meaningful information, as did the difference-score measures, with each predicting figural analogies accuracy differently and uniquely. As many of these analyses were post hoc considerations, it is thus worth considering what processes these measures are tapping into. The RSJT same-version measure was the best predictor of figural analogies accuracy overall, even though it did not interact with any of the training manipulations. Because it was comparing two identical rules, it is possible that the measure is tapping into an individual’s propensity to let surface features of the items factor into their similarity judgements. For example, consider Figure 4, which utilizes a numeracy rule. In this case, all of the items look slightly different. Some are just lines, some make a whole shape, and some include both aspects. Thus, even though they all increase an edge by two or lose an edge by one, some participants may have been accounting for surface features, with this potentially affecting their ability to solve figural analogy problems. Given that the same-version measure did predict accuracy and did also correlate with paperfolding and the Gf composite measure, it may be that a reliance on surface features is something that generally correlates with accuracy on Gf tasks. Furthermore, because the same version–different version measure did not correlate with figural analogy accuracy, Gf, WMC, or even the RSJT same version measure, but did correlate with the different-version and different-rule scores, it does appear that the RSJT same-version measure is tapping into something unique that happens when same-version comparisons are made.

The same version–different version and different rule–different version difference scores showed complementary as well as distinct findings from the RSJT same-version, different-version, and different-rule scores. The same version–different version measure was the only measure to predict trained-rule items and it predicted positively, indicating that representing different-version comparisons as less similar to same-version was beneficial, rather than what was originally hypothesized. The different rule–different version measure did support the initial findings with the RSJT different-version scores, but further specified that it was beneficial for accuracy on novel paired-rule items to represent different-version comparisons as more similar than different-rule comparisons. The same version–different version and different rule–different version measures indicate that generally treating the three comparisons as different is beneficial for accuracy. Although the same version–different version measures and the different rule–different version measures better target the difference between the three types of comparisons, it appears that all five measures produced by the RSJT measure slightly different propensities and further work will need to be conducted to better understand the differences and the similarities between all of the measures.

Although the RSJT did explain some transfer when using the same version–different version measure, it is possible that the RSJT did not explain transfer in the expected way because the measure was designed to look at the abstractness of the rule representations. Prior work has primarily looked at whether the representations include all the necessary information that is needed for transfer or mapping (Cushen and Wiley 2018), rather than looking at the quality of a representation that includes all the necessary information. Lacking information may produce a more salient effect with transfer, whereas the quality of a whole representation may matter less. Ultimately, more information is needed on the three RSJT measures to ascertain what they are actually measuring and why they are distinct from WMC and Gf, in order to determine the role of rule representations in novel problem solving and transfer.

### 8.3. Conclusions

In conclusion, individual differences in knowledge representations and WMC play independent roles in reasoning performance. WMC appears to be important for both learning and retrieving rules, as well as contributing to novel problem solving. However, beyond WMC, individual differences in knowledge representations were able to explain some aspects of performance via retrieval as well as for novel problems. The results indicate that there may be a happy medium with knowledge representations, wherein an individual will want to recognize similarities when present while also understanding distinctions between rules. Furthermore, the propensity to develop a more general or stimuli-specific knowledge representation was not explained by WMC and Gf. Thus, understanding what prompts individuals to build better representations may be key to not only understanding novel reasoning but problem solving as a whole.

## Figures and Tables

**Table 1:** Descriptive statistics.

	*n*	*M*	*SD*	Min	Max	Skew	Kurtosis	Reliability
FA Accuracy	220	11.59	4.21	4.00	24.00	0.48	0.68	0.65
RSJT SV A	115	0.69	0.15	0.26	0.96	−0.50	1.31	0.70
RSJT SV B	105	0.77	0.15	0.26	1.00	−1.18	2.51	0.76
RSJT DV A	115	0.35	0.21	−0.19	0.75	−0.55	0.83	0.68
RSJT DV B	105	0.20	0.23	−0.40	0.63	−0.25	0.47	0.71
RSJT DR A	115	−0.68	0.25	−1.00	−0.09	0.46	0.13	0.89
RSJT DR B	105	−0.68	0.26	−1.00	0.01	0.50	0.24	0.88
Operation Span	220	41.07	6.66	22.00	50.00	−0.58	0.56	0.67
Symmetry Span	220	19.70	4.90	8.00	28.00	−0.30	0.29	0.61
Paper Folding	220	11.10	3.36	4.00	19.00	−0.34	0.42	0.66
Letter Series	220	8.85	3.20	2.00	15.00	−0.07	0.23	0.76

*Note*. FA refers to figural analogies, SV refers to same version, DV refers to different version, and DR refers to different rule.

**Table 2:** Task correlations.

	1	2	3	4	5	6	7	8	9	10
1. FA Accuracy	-									
2. RSJT SV	0.29 *	-								
3. RSJT DV	0.11	0.45 *	-							
4. RSJT DR	−0.17 *	−0.14 *	0.33 *	-						
5. Operation Span	0.27 *	0.05	−0.11	−0.10	-					
6. Symmetry Span	0.37 *	0.09	0.04	−0.09	0.14 *	-				
7. Paper Folding	0.45 *	0.15 *	0.07	−0.09	0.14 *	0.30 *	-			
8. Letter Series	0.42 *	0.13	0.03	−0.08	0.17 *	0.30 *	0.32 *	-		
9. WMC Composite	0.43 *	0.10	−0.05	−0.13	0.74 *	0.77 *	0.29 *	0.31 *	-	
10. Gf Composite	0.54 *	0.17 *	0.06	−0.10	0.19 *	0.37 *	0.81 *	0.82 *	0.37 *	-

*Note.* * *p* < .05. FA refers to figural analogies, SV refers to same version, DV refers to different version, and DR refers to different rule.

**Table 3:** Predicting figural analogy accuracy with RSJT different-version scores, WMC, and Gf.

	Predictors	β	*SE*	Odds Ratio	*z*	*p*
Model 1						
	Intercept	−0.56	0.13	0.57	−4.14	<.001
	Training	0.07	0.04	1.07	1.65	0.098
	RSJT DV	0.10	0.05	1.10	1.81	0.071
	Training × RSJT DV	−0.07	0.03	0.93	−2.28	0.022
Model 2						
	Intercept	−0.59	0.13	0.55	−4.43	<.001
	Training	0.07	0.04	1.08	1.75	0.080
	RSJT DV	0.11	0.05	1.12	2.33	0.020
	WMC	0.44	0.06	1.56	7.21	<.001
	Training × RSJT DV	−0.08	0.03	0.93	−2.34	0.019
	Training × WMC	−0.06	0.04	0.94	−1.38	0.168
Model 3						
	(Intercept)	−0.59	0.13	0.55	−4.47	<.001
	Training	0.07	0.04	1.08	1.75	0.080
	RSJT DV	0.08	0.04	1.09	1.93	0.053
	WMC	0.28	0.06	1.32	4.74	<.001
	Gf	0.37	0.05	1.44	7.33	<.001
	Training × RSJT DV	−0.08	0.03	0.92	−2.50	0.012
	Training × WMC	−0.09	0.04	0.92	−2.00	0.045
	Training × Gf	0.07	0.04	1.07	1.90	0.058
Model 4 (Final Model)						
	Intercept	−0.59	0.13	0.55	−4.47	<.001
	Training	0.07	0.04	1.07	1.68	0.094
	RSJT DV	0.08	0.04	1.09	1.93	0.054
	WMC	0.28	0.06	1.32	4.66	<.001
	Gf	0.37	0.05	1.44	7.29	<.001
	Training × RSJT DV	−0.07	0.03	0.93	−2.28	0.023

*Note.* Training was coded with −1 for the novel-rules condition and 1 for the trained-rules condition. DV refers to different version.

**Table 4:** Predicting figural analogy accuracy with WMC, training, and trial.

Predictors	β	*SE*	Odds Ratio	*z*	*p*
Intercept	−0.58	0.13	0.56	−4.35	<.001
Training	0.07	0.04	1.07	1.62	0.105
WMC	0.44	0.06	1.55	7.02	<.001
Trial	0.09	0.03	1.10	3.27	0.001
Training × WMC	−0.05	0.04	0.95	−1.29	0.199
Training × Trial	0.01	0.03	1.01	0.28	0.776
Trial × WMC	0.02	0.04	1.02	0.43	0.666
Training × Trial × WMC	−0.13	0.04	0.88	−2.99	0.003

*Note.* Training was coded with −1 for the novel-rules condition and 1 for the trained-rules condition.

**Table 5:** Predicting RSJT different-version scores.

Predictors	β	*SE*	*df*	*t*	*p*
Intercept	0.00	0.18	13.36	−0.02	0.983
WMC	−0.04	0.04	216.03	−1.01	0.315
Gf	0.04	0.03	216.02	1.40	0.162

**Table 6:** Predicting figural analogy accuracy with all three RSJT measures.

Predictors	β	*SE*	Odds Ratio	*z*	*p*
Intercept	−0.58	0.14	0.56	−4.19	<.001
RSJT DV	0.04	0.07	1.05	0.69	0.492
RSJT SV	0.20	0.06	1.22	3.11	0.002
RSJT DR	−0.12	0.05	0.89	−2.16	0.031

*Note.* SV refers to same version, DV refers to different version, and DR refers to different rule.

**Table 7:** Predicting figural analogy accuracy with RSJT measures, training, and item type.

	Predictors	β	*SE*	Odds Ratio	*z*	*p*
SV Final Model						
	Intercept	−0.58	0.13	0.56	−4.30	<.001
	Training	0.06	0.04	1.06	1.48	0.140
	Item Type	−0.01	0.13	0.99	−0.10	0.919
	RSJT SV	0.24	0.05	1.27	4.53	<.001
DV Final Model						
	Intercept	−0.56	0.13	0.57	−4.13	<.001
	Training	0.07	0.04	1.07	1.72	0.085
	Item Type	−0.01	0.13	0.99	−0.11	0.912
	RSJT DV	0.09	0.05	1.10	1.76	0.079
	Training × Item Type	0.04	0.04	1.04	0.88	0.379
	RSJT DV × Training	−0.07	0.03	0.93	−2.12	0.034
	RSJT DV × Item Type	−0.04	0.03	0.96	−1.13	0.260
	RSJT DV × Training × Item Type	0.08	0.03	1.08	2.39	0.017
DR Final Model						
	Intercept	−0.55	0.13	0.57	−4.11	<.001
	Training	0.06	0.04	1.06	1.52	0.130
	Item Type	−0.01	0.13	0.99	−0.09	0.929
	RSJT DR	−0.13	0.05	0.88	−2.63	0.009

*Note.* Training was coded with −1 for the novel-rules condition and 1 for the trained-rules condition. Item type was coded with −1 for the paired-rule items and 1 for the distinct-rule items. SV refers to the same-version score, DV refers to the different-version score, and DR refers to the different-rule score.

**Table 8:** Correlations for new RSJT measures.

	1	2	3	4	5	6	7	8
1. FA Accuracy	-							
2. RSJT SV	0.30 *	-						
3. RSJT DV	0.14 *	0.51 *	-					
4. RSJT DR	−0.18 *	−0.11	0.30 *	-				
5. SV-DV	0.04	−0.08	−0.77 *	−0.65 *	-			
6. DR-DV	−0.28 *	−0.60 *	−0.42 *	0.70 *	−0.07	-		
7. WMC	0.42 *	0.11	0.00	−0.12	0.08	−0.12	-	
8. Gf	0.53 *	0.17 *	0.08	−0.10	0.03	−0.16 *	0.38 *	-

*Note.* * *p* < .05 FA refers to figural analogies, SV refers to same version, DV refers to different version, and DR refers to different rule. SV-DV refers to the same version–different version score and DR-DV refers to the different rule–different version score.

**Table 9:** Predicting figural analogy accuracy with same version–different version and different rule–different version measures and training.

Predictors	β	*SE*	Odds Ratio	*z*	*p*
(Intercept)	−0.58	0.13	0.56	−4.47	<.001
Training	0.06	0.04	1.07	1.54	0.123
SV-DV	0.00	0.04	1.00	−0.12	0.907
DR-DV	−0.13	0.04	0.88	−3.39	0.001
Gf	0.35	0.05	1.43	6.99	<.001
WMC	0.26	0.06	1.29	4.25	<.001
Training × SV-DV	0.07	0.03	1.07	2.18	0.029

*Note.* Training was coded with −1 for the novel-rules condition and 1 for the trained-rules condition. SV-DV refers to same version–different version and DR-DV refers to different rule–different version.

**Table 10:** Predicting figural analogy accuracy with same version–different version and different rule–different version measures, training, and item type.

	Predictors	β	*SE*	Odds Ratio	*z*	*p*
SV-DV Model						
	(Intercept)	−0.55	0.13	0.58	−4.12	<.001
	Training	0.06	0.04	1.06	1.49	0.136
	Item Type	−0.02	0.13	0.98	−0.13	0.895
	SV-DV	0.03	0.05	1.03	0.57	0.571
DR-DV Model						
	(Intercept)	−0.56	0.13	0.57	−4.18	<.001
	Training	0.06	0.04	1.07	1.58	0.115
	Item Type	−0.02	0.13	0.98	−0.16	0.872
	DR-DV	−0.20	0.05	0.82	−4.34	<.001
	Training × Item Type	0.04	0.04	1.04	0.94	0.345
	DR-DV × Training	0.02	0.03	1.02	0.69	0.488
	DR-DV × Item Type	0.00	0.03	1.00	−0.16	0.873
	DR-DV × Training x Item Type	−0.06	0.03	0.94	−2.25	0.025

*Note.* Training was coded with −1 for the novel-rules condition and 1 for the trained-rules condition. Item type was coded with −1 for the paired-rule items and 1 for the distinct-rule items. SV-DV refers to same version–different version and DR-DV refers to different rule–different version.

## Data Availability

Data can be accessed by contacting the authors of the study.

## Notes

1 To address concerns that composite variables for WMC and Gf may not be appropriate, several models were checked with each of the individual indicators for these variables, rather than the composite variables. In the WMC x Novel/Learned x Trial interaction, using Operation Span instead of the composite moved the *p*-value to *p* = .07, rather than being significant, while the interaction remained significant using only Symmetry Span. In the Representation Scores x Novel/Learning + WMC + Gf analysis, using the individual Gf tasks moved the main effect of DV representation scores from non-significant to significant. When predicting SV-DV training, changes from composites to individual measures changed nothing. As these are minor differences, composites were used throughout the paper for analyses.

2 Only 3 participants failed to use the highest marking on the scale (100) for any of their scores; all 3 instead used 95 as their top score. A total of 29 participants put their lowest raw score as something other than 0. For 2, the lowest score was 25, for 2 others it was 20, and for 4 it was 15. All of the rest used 5 or 10 as their lowest number. Because 86% of the sample used the full scale, it is not anticipated that this caused substantial changes to the analyses.

## Appendix A

**Table A1:** Figural analogy rules.

Rule	Version	Description	Set
Rotation	1	Object rotates 45 degrees clockwise	A
Rotation	2	Object rotates 90 degrees clockwise	A
Color Change	1	Object fill changes from either dark fill or white fill	A
Color Change	2	Object fill changes from one pattern to another pattern	A
Shift	1	Object moves diagonally to another location	A
Shift	2	Object moves vertically to another location	A
Merge	1	Two objects combine, such as a puzzle piece or slice of pie, creating a whole shape	A
Merge	2	Two objects combine, such as a puzzle piece or slice of pie, creating a shape where the added piece juts out	A
Pattern	1	The number of notches in the object determines the number of sides	A
Pattern	2	The color of the object (e.g., black or white) determines the type of object	A
Duplicate	1	The object is duplicated, resulting in 2 of the same object	A
Duplicate	2	The object is duplicated, resulting in 3 of the same object	A
Count	1	The number of sides increases by 2	A
Count	2	The number of sides decreases by 1	A
Sharpness	1	The corners of the object go from sharp to round	B
Sharpness	2	The edges of the object go from straight to curved	B
Location	1	Three objects in a straight line move to create a diagonal line	B
Location	2	Three objects in a straight line move to make a triangle shape	B
Size	1	Object increases in size	B
Size	2	Object decreases in size	B
Invert	1	Notches in an object change to instead stick out of the object	B
Invert	2	Parts of an object that jut out change to become notches	B
Reflect	1	Object reflects over an imaginary *x* axis	B
Reflect	2	Object reflects over an imaginary *y* axis	B
Swap	1	Two objects swap locations in within a grid-like design	B
Swap	2	Two objects swap locations but one object was inside the other object	B
Divide	1	A fourth of an object is removed	B
Divide	2	A third of an object is removed	B

**Table A2:** Post hoc comparisons: RSJT different-version × training × item type.

	Predictors	β	*SE*	Odds Ratio	*z*	*p*
Trained Items Only						
	(Intercept)	−0.49	0.14	0.61	−3.48	0.001
	RSJT DV	0.04	0.06	1.04	0.59	0.553
	Item Type	0.02	0.14	1.02	0.18	0.855
	RSJT DV × Item Type	0.05	0.05	1.05	1.01	0.314
Novel Items Only						
	(Intercept)	−0.63	0.14	0.53	−4.44	0.000
	RSJT DV	0.18	0.06	1.19	2.79	0.005
	Item Type	−0.05	0.14	0.95	−0.40	0.690
	RSJT DV × Item Type	−0.11	0.05	0.90	−2.33	0.020
Distinct-Rule Items Only						
	(Intercept)	−0.57	0.18	0.56	−3.27	0.001
	RSJT DV	0.06	0.06	1.06	0.96	0.339
	Training	0.11	0.06	1.11	1.72	0.086
	RSJT DV × Training	0.01	0.05	1.01	0.14	0.892
Paired-Rule Items Only						
	(Intercept)	−0.54	0.19	0.58	−2.80	0.005
	RSJT DV	0.14	0.06	1.14	2.30	0.021
	Training	0.03	0.05	1.03	0.63	0.530
	RSJT DV × Training	−0.14	0.05	0.87	−3.20	0.001

*Note.* Training was coded with −1 for the novel-rules condition and 1 for the trained-rules condition. Item type was coded with −1 for the paired-rule items and 1 for the distinct-rule items. DV refers to different version.

**Table A3:** Post hoc comparisons: WMC × training × trial.

	Predictors	β	*SE*	Odds Ratio	*z*	*p*
Trained Items Only						
	(Intercept)	−0.52	0.14	0.60	−3.72	<.001
	WMC	0.39	0.08	1.47	5.13	<.001
	Trial	0.10	0.04	1.11	2.57	0.010
	WMC × Trial	−0.11	0.06	0.90	−1.81	0.070
Trained Items Only						
	(Intercept)	−0.64	0.14	0.52	−4.60	<.001
	WMC	0.38	0.07	1.46	5.10	<.001
	Trial	0.09	0.04	1.09	2.33	0.020
Novel Items Only						
	(Intercept)	−0.64	0.14	0.52	−4.60	<.001
	WMC	0.49	0.07	1.64	6.58	<.001
	Trial	0.08	0.04	1.08	1.91	0.056
	WMC × Trial	0.14	0.06	1.15	2.30	0.021

**Table A4:** Post hoc comparisons: RSJT different-rule × Gf × training.

	Predictors	β	*SE*	Odds Ratio	*z*	*p*
Trained Items Only						
	(Intercept)	−0.51	0.14	0.60	−3.75	<.001
	RSJT DR	−0.11	0.05	0.90	−2.15	0.031
	Gf	0.48	0.06	1.61	8.17	<.001
	RSJT DR × Gf	−0.06	0.06	0.94	−0.97	0.330
Novel Items Only						
	(Intercept)	−0.62	0.14	0.54	−4.45	<.001
	RSJT DR	−0.07	0.05	0.93	−1.36	0.175
	Gf	0.43	0.06	1.53	6.86	<.001
	RSJT DR × Gf	0.10	0.06	1.10	1.55	0.122
High Gf Only						
	(Intercept)	−0.29	0.16	0.75	−1.83	0.067
	RSJT DR	−0.12	0.07	0.89	−1.81	0.070
	Training	0.11	0.05	1.11	2.24	0.025
	RSJT DR × Training	−0.08	0.04	0.92	−1.96	0.050
Low Gf Only						
	(Intercept)	−0.83	0.12	0.44	−7.14	<.001
	RSJT DR	−0.12	0.06	0.89	−1.98	0.048
	Training	0.00	0.05	1.00	−0.02	0.987
	RSJT DR × Training	0.05	0.04	1.05	1.12	0.263
Low Gf Only						
	(Intercept)	−0.83	0.12	0.44	−7.14	<.001
	RSJT DR	−0.12	0.06	0.89	−2.06	0.040
	Training	0.00	0.05	1.00	−0.06	0.952

*Note.* Training was coded with −1 for the novel-rules condition and 1 for the trained-rules condition. DR refers to different rule.

**Table A5:** Post hoc comparisons: different-rule–different-version × training × item type.

	Predictors	β	*SE*	Odds Ratio	*z*	*p*
Trained Items Only						
	(Intercept)	−0.49	0.14	0.61	−3.56	<.001
	DR-DV	−0.18	0.05	0.84	−3.30	0.001
	Item Type	0.02	0.13	1.02	0.14	0.886
	DR-DV × Item Type	−0.07	0.04	0.93	−1.71	0.087
Novel Items Only						
	(Intercept)	−0.62	0.14	0.54	−4.45	<.001
	DR-DV	−0.22	0.05	0.80	−4.07	<.001
	Item Type	−0.06	0.13	0.94	−0.45	0.653
	DR-DV × Item Type	0.06	0.04	1.06	1.46	0.143
Distinct-Rule Items Only						
	(Intercept)	−0.58	0.17	0.56	−3.35	0.001
	DR-DV	−0.20	0.05	0.82	−3.67	<.001
	Training	0.10	0.06	1.11	1.66	0.098
	DR-DV × Training	−0.04	0.04	0.96	−1.05	0.293
Paired-Rule Items Only						
	(Intercept)	−0.53	0.19	0.59	−2.80	0.005
	DR-DV	−0.19	0.05	0.83	−3.76	<.001
	Training	0.03	0.05	1.03	0.48	0.630
	DR-DV × Training	0.08	0.04	1.09	2.20	0.028

*Note.* Training was coded with −1 for the novel-rules condition and 1 for the trained-rules condition. Item type was coded with −1 for the paired-rule items and 1 for the distinct-rule items. DR-DV refers to different rule–different version.
